# Analysis of the dynamics of temporal relationships of neural activities using optical imaging data

**DOI:** 10.1007/s10827-016-0630-8

**Published:** 2016-10-24

**Authors:** Jannetta S. Steyn, Peter Andras

**Affiliations:** 1 Bioinformatics Support Unit, Newcastle University, Newcastle upon Tyne, NE1 7RU UK; 2 School of Computing and Mathematics, Keele University, Keele, ST5 5BG UK

**Keywords:** Stomatogastric ganglion, Voltage sensitive dye, Computational analysis, Neuron-scale imaging, Synchronisation

## Abstract

The temporal relationship between the activities of neurons in biological neural systems is critically important for the correct delivery of the functionality of these systems. Fine measurement of temporal relationships of neural activities using micro-electrodes is possible but this approach is very limited due to spatial constraints in the context of physiologically valid settings of neural systems. Optical imaging with voltage-sensitive dyes or calcium dyes can provide data about the activity patterns of many neurons in physiologically valid settings, but the data is relatively noisy. Here we propose a numerical methodology for the analysis of optical neuro-imaging data that allows robust analysis of the dynamics of temporal relationships of neural activities. We provide a detailed description of the methodology and we also assess its robustness. The proposed methodology is applied to analyse the relationship between the activity patterns of PY neurons in the crab stomatogastric ganglion. We show for the first time in a physiologically valid setting that as expected on the basis of earlier results of single neuron recordings exposure to dopamine de-synchronises the activity of these neurons. We also discuss the wider implications and application of the proposed methodology.

## Introduction

The dynamics of the temporal relationship of the activities of neurons forming neural circuits is critically important for the flexible and adaptive delivery of the functionality of these circuits (note: in this paper we use the term neural activity to mean the variation of the membrane potential of a neuron, including spikes, sub-threshold membrane potential changes, and any other membrane potential changes; this term is not used to mean spike count or some other kind of statistical summary metric of the activity of a neuron) (Harris-Warrick et al. 1992; Fdez Galán et al. 2004; Hill et al. 2012; Bruno et al. 2015). For example, switching between synchronised and de-synchronised patterns of activity of neurons forming functional circuits in the hippocampus plays a fundamental role in memory formation, maintenance and recall in vertebrate brains (Axmacher et al. 2006; Robbe et al. 2006). In the case of epilepsy a switch to excessive synchronisation of neural activities breaks down the functionality of many neural circuits and neural systems formed by them (Feldt Muldoon et al. 2013; Engel et al. 2013). Recently, it has been shown that the fine timing of inputs to different parts of the dendritic tree of neurons in the visual cortex of mammals determines the spatio-temporal preferences of these parts of the neuron (Chen et al. 2013). The combination of the preferences determines the actual receptive field of the neuron. In general, both relatively simple and complex changes in the temporal relationship of neural activities can play a critical role in the delivery of the functionality of neural circuits.

Until relatively recently the recording of many synaptically connected neurons at individual neuron resolution, i.e. at neuron-scale, was not possible in the context of physiologically realistic conditions e.g. the use of individual micro-electrodes implies significant spatial constraints limiting the number of recordable neurons (Miller 1987). While multi-electrode arrays allow recording of many individual neurons in artificially created cell culture (Potter and DeMarse 2001; Spira and Hai 2013), the activity of neurons in such context is not truly comparable to the activity of neurons in real physiological conditions. In other settings when multi-electrode array or multiple multi-electrodes (e.g. tetrodes) are used to record many neurons form brains or brain slices in physiological conditions the connectivity between the recorded neurons is usually not known (Guitchounts et al. 2013; Scholvin et al. 2015; Santos et al. 2012). Multi-electrode arrays (Meyer et al. 2016) or high-resolution surface EEG (Ohl et al. 2001) may be also applied to in-vivo recording from the surface of an intact brain. However, these methods record local field potentials from the surface of the brain which are a mixture of signals originating from many neurons, making it very difficult and often impossible to read out the activity of identifiable individual neurons. The impact of this is that a large part of the work on neuron resolution dynamics of neural circuits remained mostly theoretical (Schneidman et al. 2006; Shlens et al. 2006; Paninski et al. 2010).

Currently used techniques of optical recording of neural activity using voltage-sensitive dyes and calcium dyes allow high spatio-temporal resolution recording of the activity of many neurons, making possible the study of the dynamics of temporal relationships of neural activities in biological neural circuits (Canepari and Zecevic 2010). While many applications of these techniques are used to record many neurons that are not necessarily directly coupled synaptically (Ea et al. 2009; Rothschild et al. 2010), it has been shown that these methods can also be applied successfully to a range of biological neural systems to record the activity of many synaptically coupled neurons simultaneously. These techniques have been applied to analyse the functionality of neurons in leech ganglia (Briggman et al. 2010), to study the dynamical assignment of functional roles to neurons in snail ganglia (Hill et al. 2012; Bruno et al. 2015), to record almost simultaneously the activity of all neurons in the brain of the zebra fish embryo (Ahrens et al. 2012), to analyse the activity of neurons in intestinal neural ganglia in guinea pigs (Obaid et al. 1999), and to study the activity of synaptically coupled neurons in the stomatogastric ganglion of crabs (Stein et al. 2011; Städele et al. 2012). However, it should be noted that usually the recorded data is quite noisy, potentially making its analysis difficult.

Here we address the issue of analysis of such optical imaging data for the purpose of understanding the dynamics of temporal relationship of the activities of individual neurons. Our method relies on the identification of a few key features of the activity patterns of individual neurons, which can be estimated sufficiently robustly from the recorded noisy data. For example, consider the case of neurons which spike during depolarisation plateaus and the recording of the neuron starting before a such depolarisation plateau and ending after the plateau. The numerically calculated local maximum upward slope point of the recorded activity approximates the start of the depolarisation plateau. The calculated minimum downward slope point of the recorded activity approximates the end of the depolarisation plateau. Having the timings of the identified key features of the neural activity patterns we can use these to estimate the changes in the temporal relationships of neural activities and thus the dynamics of the temporal relationships between neural activities. The method that we describe applies in particular to bursts of spikes and the estimation of temporal dynamics of burst periods of multiple neurons. The method is most applicable to invertebrate neurons with high depolarisation plateaus, but we also indicate how to adapt it to vertebrate neurons with lower depolarisation plateaus as well.

We apply the proposed data analysis method to neurons recorded in the crab stomatogastric ganglion. According to earlier results about the impact of dopamine on individual pyloric constrictor (PY) neurons it can be expected that dopamine exposure causes the de-synchronisation of the activity of these neurons (Johnson et al. 1993, 1994; Ayali et al. 1998). We analysed and quantified the impact of dopamine on the temporal relationship between the activity patterns of PY neurons. Our results show that as expected there is a statistically significantly measurable de-synchronisation effect in the case of the considered PY neurons in general. We note that this is the first report of this effect in physiologically valid conditions, as earlier results were obtained by recording single neurons following application of neurotoxic substances to achieve pharmacological isolation of them.

The rest of the paper is structured as follows. First we review the relevant background. Then we describe the proposed methodology in detail. Next we describe the application of the methodology to voltage-sensitive dye recording of the activity of PY neurons in the crab stomatogastric ganglion. Finally we discuss the implications of the presented work and draw the conclusions.

## Background

### Neuron-scale temporal dynamics

Synchronisation of the activity of neurons is a common pattern across biological neural systems and plays a critical role in the functionality of many neural circuits (Axmacher et al. 2006; Robbe et al. 2006; Feldt Muldoon et al. 2013). The transition from the non-synchronised to the synchronised state of a number of individual neurons is thus perhaps the most commonly found dynamical behaviour of the relative activities of individual neurons. For example, in the hippocampus the formation of new memories is supported by the temporary synchronisation of the activity of blocks of neurons (Robbe et al. 2006). Such temporarily synchronised activity of hippocampal neurons also plays a key role in the maintenance and recall of memories (Axmacher et al. 2006). The role of temporary synchronisation of neural activities has been investigated extensively in theoretical neuroscience (e.g. synfire chains) (Abeles et al. 2004). In many theoretical models of neural circuits temporary synchronisation of neural activities is at the core of the functionality of the model circuit (Abeles et al. 2004; Ikegaya et al. 2004; Burkitt and Clark 1999).

De-synchronisation of neural activities is at least as important for normal functioning of neural circuits as synchronisation of neural activities (Engel et al. 2013; Feldt Muldoon et al. 2013). Lack of de-synchronisation and excessive synchronisation of neural activities is the underlying mechanism of epileptic seizures in vertebrates (Feldt Muldoon et al. 2013). Following the synchronisation of neurons in the hippocampus their de-synchronisation is required in order to support the formation of new memories. De-synchronisation of neural activities following brief synchronous activity also happens in many areas of the mammalian cortex where synchronisation of neurons may represent temporary binding of features of animal actions and perceptions and de-synchronisation makes the neural circuits ready to process new information related to new perceptions and actions of the animal (Raffone and Wolters 2001; Finger and König 2013).

The fine temporal patterning of inputs to neurons plays a major role in the functioning of single neurons and of neural circuits made of these neurons (Gutierrez et al. 2013; Marder 2012). For example,in the cerebellum the Purkinje cells may receive thousands of inputs in appropriate temporal ordering making them able to compute their activity required for the fine tuning control of the musculature of the animal (Feldman 2012; Kawamura et al. 2013). Recently it has been shown that the fine temporal pattern of inputs differently tunes the activity of parts of the dendritic trees of pyramidal neurons in the visual cortex and the combination of these activities determines the actual receptive field features of the neuron (Chen et al. 2013). Thus, fine changes in the temporal relationships in the activities of neurons that provide inputs to such neurons may change the receptive field properties, of the neurons that receive this patterned input, considerably.

In the context of several biological neural systems it has been shown that neurons may change their functional role within some range and such changes are indicated by dynamics of the temporal relationships of neural activities (Hill et al. 2012; Marder 2012; Gutierrez et al. 2013). In the case of hippocampal place cells, the same neuron may represent different parts of the spatial environment of the animal depending on the changes of the environment (Hartley et al. 2014). Thus the same neuron may be active in different functional circuits depending on changes to of the spatial environment of the animal. Similarly, it has been shown that some neurons in the swim controlling central pattern generators in the dorsal cerebral ganglia of *Tritonia diomedea* can switch their role by participating in the control of different phases of the swim cycles (Hill et al. 2012). These role changes of neurons are achieved through dynamic re-arrangement of the temporal relationships between the activities of the involved neurons.

### Neuron-scale recording of multi-neuron activity

Neuron-scale recording of the activity of a number of neurons is possible using micro-electrodes (Harris-Warrick et al. 1992; Spira and Hai 2013), however the number of simultaneously recorded neurons is limited by the physical size of electrode manipulators. For example, in the case of invertebrate ganglia with large neurons it is possible to record 4 - 5 neurons simultaneously (Miller 1987). A larger number of neurons can be recorded simultaneously using micro-electrode arrays combined with neuronal cell cultures (Spira and Hai 2013; Potter and DeMarse 2001). However in this latter case the neurons are not in any physiologically valid setting and the interpretation of the recordings cannot be easily related to the functionality of biological neural circuits.

Since the late 1980s it has been demonstrated that optical imaging using voltage-sensitive dyes and calcium dyes allows the recording of the activity of many neurons simultaneously in biological neural systems in their physiological settings (Canepari and Zecevic 2010). These techniques have been applied to snail ganglia (Hill et al. 2012; Bruno et al. 2015), leech ganglia(Briggman et al. 2010), and guinea pig intestinal ganglia (Obaid et al. 1999) to study the activity of individual neurons in the context of the neural circuits in which they participate. More recently voltage-sensitive dye imaging has been used to record and analyse the activity of neurons with known synaptic connections in the stomatogastric ganglion of crabs (Stein et al. 2011; Städele et al. 2012), to analyse the role switching behaviour of neurons in snail ganglia (Hill et al. 2012), and to establish the functional role of individual neurons in leech ganglia (Briggman et al. 2010).

A recent variant of the calcium imaging technique allows the individual recording of most neurons in the brain of zebrafish embryos using light-sheet microscopy and mutant zebrafish that express fluorescent calcium indicator molecules in their neurons (Ahrens et al. 2012). Although the temporal resolution of this method is insufficient for very fine analysis of the temporal relationships of neural activities (the imaging happens at 0.8 Hz) the data is sufficiently good to analyse larger scale changes in the organisation of neural activity patterns in various parts of the zebrafish brain (Ahrens et al. 2012).

Another recent approach uses laser scanning microscopy combined with rapid changes of the three-dimensional position where the laser beam points within the neural tissue allowing the rapid recording of many individual neurons at various positions in the three-dimensional arrangement of the neural tissue (Fernández-Alfonso et al. 2014). This method makes possible the recording of up to a couple of hundred of individual neurons at high temporal resolution. However, the applications of this methodology have been so far in the context of higher neural systems (e.g. parts of the cortex of mammals), where there are very many neurons and the connectivity of the neurons is not known in advance, making the functional interpretation of the recordings somewhat complicated.

## Proposed methodology

**Fig. 1:**
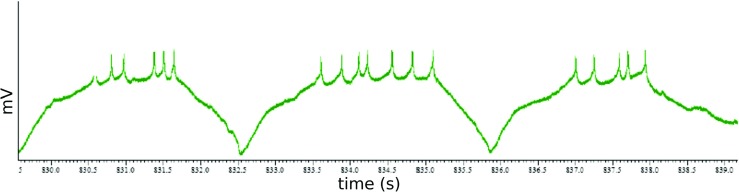
Intracellular recording of a neuron from the crab stomatogastric ganglion. The spiking of the neurons happen during the depolarisation plateaus. The horizontal axis is time, the vertical axis is voltage in arbitrary units

The activity of neurons participating in biological neural circuits follows various patterns. Some neurons are silent most of the time balancing around their resting potential and fire rarely single spikes or a few spikes, for example some cortical neurons in mammals behave in this way (Brumberg et al. 2000). Other neurons generate bursts of activity periodically, for example invertebrate neurons that form central pattern generators(Harris-Warrick et al. 1992). One relatively common feature of the various neural activities is that generally the spiking of neurons (especially multiple spikes) happens on the top of a depolarization plateau measured in the soma (see Fig. 1). In some cases the amplitude of membrane potential difference deviations during the spikes is larger (possibly much larger) than the amplitude of depolarization for the plateau (Brumberg et al. 2000), in other cases the depolarization amplitude of the plateau can be of comparable size or even larger than the amplitude of membrane potential difference changes during spikes (Harris-Warrick et al. 1992). In general the change in the relative temporal ordering of the activity of multiple neurons is represented by changes in the relative timing of individual spikes or bursts of spikes generated by these neurons. Thus, considering that spikes and bursts of spikes happen usually on the top of an depolarization activity plateau, the temporal dynamics of relative activities of neurons must be also reflected by the dynamics of the relative timing of such activity plateaus of these neurons.

In the case of micro-electrode intra-cellular recording of neurons individual spikes, even as part of bursts of spikes, can be distinguished easily. In the case of optical imaging recording of neural activities this is often not the case due to the inherent noise of imaging data acquisition. This means that relying on the determination of spike and burst times of individual neurons is relatively difficult with optical imaging data. Consequently, in the case of optical imaging data, these neural activity markers are relatively unreliable for the estimation of the temporal relationships dynamics of neural activities.

**Fig. 2:**
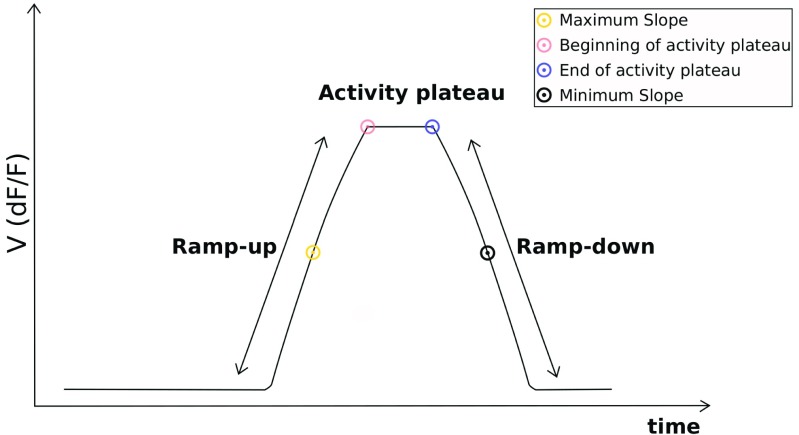
Typical activity profile of a neuron. The spiking happens during the activity plateau, which is preceded by the ramp-up phase and followed by the ramp-down phase. The vertical axis shows the membrane potential of the neuron

Here we propose to use the timing of the activity plateaus of neurons for the estimation of the dynamics of the temporal relationship of their activities. The heuristic analysis that we propose works off-line, following the recording of the activity of the neurons. In order to use activity plateaus for this purpose we need to define a set of salient features of the neural activity patterns that can be determined robustly using the noisy optical imaging data. Given that in general the activity plateaus are preceded by a ramp-up phase and are followed by a ramp-down phase of the membrane potential in the soma of the neuron, the salient features of neural activity profile that we chose are indicators of the timing of the ramp-up, ramp-down and the beginning and ending of the plateau itself (see Fig. 2 for an illustration).

To find the timing of the ramp-up phase we numerically determine the time point for which the upward slope of the neural activity profile is maximal during an appropriately chosen time interval that lasts for around the usual duration of the measured ramp-up phases. This point is expected to be around the mid-point of the ramp-up phase. To find the maximum upward slope point (or maximum slope point, given that the upward slope is a positive slope) we calculate for each time step the slope of the best linear approximation of the data points representing the neuron’s activity profile for an appropriately chosen time window centred on the time of the given time step. First the raw values of the measured imaging data are smoothed appropriately to reduce the impact of random noise in the data. Assuming that *x*_*t*_ are the smoothed measured values of the neural activity at recording time step t *t*, this means that we consider time intervals of 2*τ* + 1 measurement time units and calculate the local slope approximation *m*_*t*_ such that

1 $$(m_{t},b_{t}) =\underset{m,b}{\arg \min} \sum\limits_{u =t -\tau}^{t +\tau} (x_{t} -m \cdot (u -t +\tau ) -b)^{2}  $$

where *m* are slope values and *b* are bias values in linear approximations of *x*_*t*_.

The maximum slope point for a time interval [*T*_1_, *T*_2_], measured in units of recording time steps, is the point on the activity profile of the neuron corresponding to the time point *t* ^∗^ for which

2 $$m_{t^{\ast} } =\max_{t \in [T_{1},T_{2}]}m_{t} $$

**Fig. 3:**
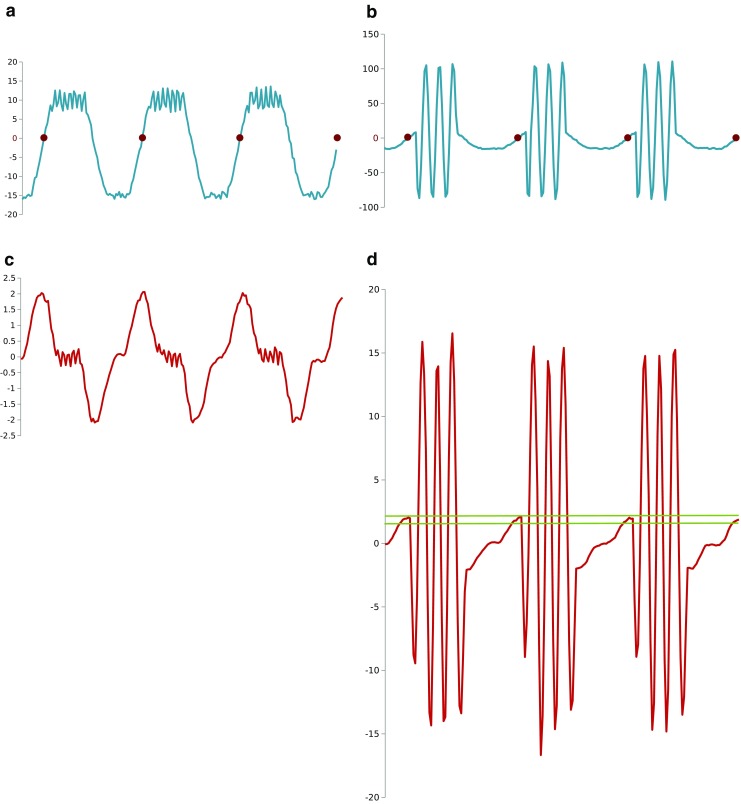
**a** Maximum slope points (as *dots*) calculated for simulated neural activity having plateau potential difference that is much larger than the potential difference corresponding to spiking activity; **b** The calculated local slope values for the data shown in **a**); **c** Maximum slope points (as *dots*) calculated for simulated neural activity having plateau potential difference that is much smaller than the potential difference corresponding to spiking activity; **d** The calculated local slope values for the data shown in **c)**. The *horizontal lines* in **d)** indicate the range of local slope values that are considered for maximum slope point identification. The horizontal axis is always time, the vertical axis represents the voltage in **a)** and **c)** in arbitrary units and the local slope value in **b)** and **d)**

If the time interval [*T*_1_, *T*_2_] is chosen such that *T*_2_ − *T*_1_ is approximately the usual time length of the ramp up phase (measured in units of recording time steps) and *τ* is chosen appropriately (e.g. *τ* = (*T*_2_ − *T*_1_)/2 or slightly less), then it can be expected that the above calculation will find the maximum slope point of the neural activity profile corresponding to the time interval [*T*_1_, *T*_2_]. If the chosen time interval is such that during this time interval the neuron’s activity profile follows a ramp-up phase, the maximum slope point that we find is likely to indicate the midpoint of the ramp-up phase. If the chosen interval is such that the activity profile of the neuron for this interval does not match a ramp-up phase the maximum slope point that we find will not indicate the mid-point of a ramp-up phase, and we call these spurious maximum slope points. To distinguish between maximum slope points which indicate valid mid-points of ramp-up phases and those which do not, we have to consider the value ranges of the calculated maximum slope values for many considered time intervals. If the membrane potential variation associated with the ramp-up phase is larger than the membrane potential variation associated with spikes measured in the neuron soma, the slope values for valid maximum slope points will be much larger than the slope values calculated for spurious maximum slope points. In general spurious maximum slope points calculated for periods of relative silence of neural activity will have small maximum slope values associated to them (possibly very close to zero). If the soma membrane potential variations associated with spikes are larger than the soma membrane potential variation during the ramp-up phase, some spurious maximum slope points may have larger slope values associated with them than the slope values calculated for valid maximum slope points. In such cases we have to rely on setting the appropriate and sufficiently narrow value interval for valid maximum slope values based on the analysis of the experimental data. Figure 3 presents synthetic examples of these two cases demonstrating the determination of the maximum slope points.

For the timing of the ramp-down phase we determine the time point with the maximal downward slope of the neural activity profile. We proceed in a similar manner as in the case of the determination of the maximum slope point. In the case of the ramp-down phase the general expectation is that the minimum slope (equivalent of the maximum downward slope) point is around the middle of the ramp-down phase. To find the minimum slope point we use again the calculation for each time step of the slope of the best linear approximation of the data points representing the neuron’s activity profile for an appropriate time window centred on the given time step (see Eq. (1)). The minimum slope point for a time interval [*T*_1_, *T*_2_] measured in units of recording time steps is the point on the activity profile of the neuron corresponding to the time point *t* ^∗∗^ for which

3 $$m_{t^{\ast \ast} } =\min_{t \in [T_{1},T_{2}]}m_{t}  $$

As stated previously, for appropriately chosen *T*_2_ − *T*_1_ and *τ* it can be expected that Eq. (3) finds the minimum slope point of the neural activity profile corresponding to the time interval [*T*_1_, *T*_2_]. If the chosen interval is such that the activity profile of the neuron for this interval does not match a ramp-down phase the minimum slope point that we find will not indicate the mid-point of a ramp-down phase, and we call these a spurious minimum slope points similarly to spurious maximum slope points. As in the case of maximum slope points, if the membrane potential change associated with the ramp-down phase is considerably larger than the soma membrane potential change associated with spikes, the valid minimum slope points will be significantly smaller than the spurious minimum slope points, which are expected to have values close to zero. If the membrane potential changes in the soma associated with spikes are larger than the membrane potential change of the ramp-down phase, the determination of the valid minimum slope points relies on the experimental determination of the acceptability range of valid minimum slope values and those minimum slope points are considered valid for which the associated slope value is in this acceptability range.

In the case of neurons with large change of membrane potential difference during ramp-up and ramp-down phases and relatively small changes of the membrane potential difference during the spikes the calculation of local slope approximation also allows the estimation of the beginning and the end of the activity plateau. This cannot be done reliably for neurons where the membrane potential difference changes in the soma during spiking are much larger than the changes during the ramp-up and ramp-down phases (see Fig. 3c and d).

To find the estimated points for the beginning and the end of the activity plateau we consider the local forward and backward slopes of the neural activity. The local forward slope at a time point is the slope of the best linear approximation of the neural activity starting from that time point and for some time period forward. It is expected that the local forward slope gets close to zero around the start of activity plateau, given that the soma membrane potential difference variations related to spikes are relatively small, and that the local forward slope is considerably positive for time points before the start of the activity plateau. Similarly, the local backward slope at a time point is the slope of the best linear approximation of the neural activity over some time period ending at this time point. In general, it can be expected that the local backward slope is close to zero for time points on the activity plateau and becomes considerably negative as the activity of the neurons goes into the ramp-down phase. So, the end of the activity plateau is indicated by the last time point where the local backward slope is close to zero. We calculate the estimated local forward and backward slope, ${m_{t}^{f}}$ and ${m_{t}^{b}}$, respectively, as follows

4 $$({m_{t}^{f}},{b_{t}^{f}}) =\frac{argmin}{m,b} \sum\limits_{u =t}^{t +2 \tau} (x_{t} -m \cdot (u -t +\tau ) -b)^{2}  $$

5 $$({m_{t}^{b}},{b_{t}^{b}}) =\frac{argmin}{m,b} \sum\limits_{u =t -2 \tau}^{t}(x_{t} -m \cdot (u -t +\tau ) -b)^{2}  $$

We look for points on the activity profile of the neuron for which the calculated local forward and backward slope values are close to zero. The acceptable range of close to zero values may be determined on a case-by-case basis examining the calculated slope values or in principle we may choose the acceptability range as [−*𝜖*⋅*m*_*max*_, *𝜖*⋅*m*_*max*_], where *m*_*max*_ is the maximal absolute value of the calculated slope values associated with maximum and minimum slope points and *𝜖* is a small number, for example *𝜖* = 0.1. We call this range of values the zero value range and denote it as [−*z* ^∗^,*z* ^∗^]. Following the finding of all points with forward and backward slope values within the zero value range we determine the first of these that follows a maximum slope point and the last that precedes the minimum slope point, these two points will be the estimates of the beginning and the end, respectively, of the activity plateau of the neuron. In formal terms we determine

6 $$T^{z,f} =\{t\vert {m_{t}^{f}} \in [ -z^{\ast} ,z^{\ast} ]\}  $$

7 $$T^{z,b} =\{t\vert {m_{t}^{b}} \in [ -z^{\ast} ,z^{\ast} ]\}  $$

then ${t_{b}^{0}}$ and ${t_{e}^{0}}$ are determined such that:

8 $${t_{b}^{0}} >t^{\ast} ,{t_{b}^{0}} \in T^{z,f},{t_{b}^{0}} \leq t, \forall t \in T^{z,f},t >t^{\ast}  $$

9 $${t_{e}^{0}} <t^{\ast \ast} ,{t_{e}^{0}} \in T^{z,b},{t_{e}^{0}} \geq t, \forall t \in T^{z,b},t <t^{ \ast \ast}  $$

**Fig. 4:**
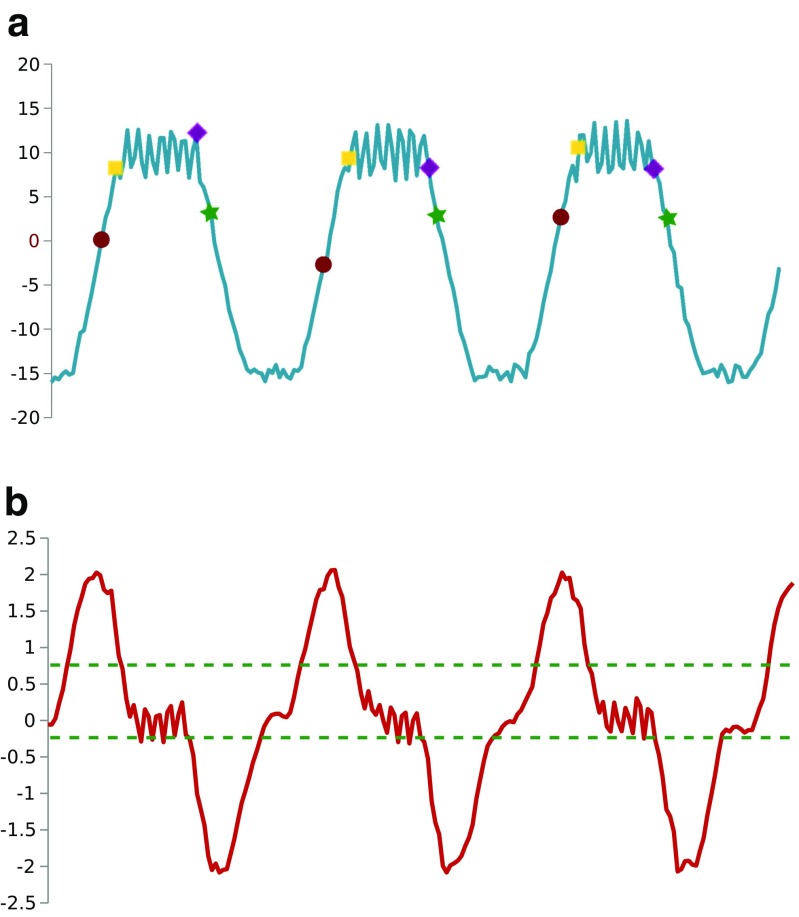
**a** Simulated activity of a neuron. The **calculated maximum slopes** are shown as *red dots* and the **calculated minimum slopes** are shown as *green dots*. The beginning and end points of activity plateaus are shown with *yellow squares* and *purple diamonds* respectively; **b** The *thin green lines* indicate the value band that is considered to correspond to the activity plateau following the maximum slope point. The horizontal axis is time in both cases, while the vertical axis represents voltage in **(a)** in arbitrary units and the calculated local slope value in **(b)**

The beginning and end of the activity plateau will be the points corresponding to the time steps ${t_{b}^{0}}$ and ${t_{e}^{0}}$, respectively. In general, we expect to be able to determine an activity plateau for each consecutive pair of maximal and minimal slope points. Figure 4 exemplifies the determination of maximum and minimum slope points and the beginning and end points of activity plateaus using synthetic data for a neuron with large membrane potential difference changes associated with the ramp-up and ramp-down phases and relatively small such changes in the soma associated with spikes. We note that the reliability of estimation of the maximal and minimal slope points is better than the estimation of the beginning and end points of the activity plateau, because the latter depend on the choice of the acceptability range of close to zero values. However, estimating all four above defined salient features of the neural activity profile, if possible, provides more information for the analysis of the dynamics of the temporal relationships of the activity multiple neurons.

Following the determination of maximum and minimum slope points and possibly of the beginning and end points of activity plateaus for multiple neurons recorded simultaneously we can use the timing of these points to analyse the changes in the temporal relationship of the activities of the recorded neurons. Depending on the number of the kinds of salient points that we can determine we get multiple estimates about the observable temporal features of the joint activity of the considered neurons. For example, the average time difference between maximum slope points of two rhythmically active neurons indicates the temporal difference between the activation of these neurons, while the average time difference between minimum slope points of the same neurons indicates the temporal difference between the inactivation of these neurons. Phase locking between the neurons is indicated by small standard deviation of the calculated temporal differences between matching maximum slope or minimum slope points of the neurons and the relaxation of phase locking is implied by an increase of the standard deviation for example following of exposure to a neuromodulator.

To assess the robustness of the above proposed calculations let us consider that $x_{t} =\tilde {x}_{t} +z_{t}$, where $\tilde {x}_{t}$ is the true value of the membrane potential difference and *z*_*t*_ is an additive normal noise with zero mean and *σ* standard deviation. Considering the formula in Eq. (1) for the local slope (a similar approach applies for the local forward and backward slope as well), following algebraic manipulation we find that

10 $$m_{t} =\frac{3}{\tau (\tau +1) (2 \tau +1)} \cdot \sum\limits_{u =t -\tau}^{t +\tau} (u -t) \cdot x_{u}  $$

Considering the composition of *x*_*t*_ leads to:

11 $$m_{t} =\tilde{m}_{t} +\mu_{t}  $$

where $\tilde {m}_{t}$ is the correct local slope value and *μ*_*t*_ is an additive noise in the estimate of the by *m*_*t*_. For *μ*_*t*_ we get that:

12 $$\mu_{t} =\frac{3}{\tau (\tau +1) (2 \tau +1)} \cdot \sum\limits_{u = -\tau}^{\tau} u \cdot z_{u +t}  $$

13 $$\sigma_{\mu_{t}}^{2} =\frac{9}{(\tau (\tau +1) (2 \tau +1))^{2}} \cdot \sum\limits_{u = -\tau}^{\tau} u^{2} \cdot \sigma^{2} =\frac{3 \sigma^{2}}{\tau (\tau +1) (2 \tau +2)}  $$

and

14 $$\bar{\mu_{t}} =0  $$

where *σ*_*μt*_ is the standard deviation and $\bar {\mu _{t}}$ is the mean value of *μ*_*t*_.

Thus the additive noise in the estimates of the local slope follows a normal distribution with zero mean and standard deviation equal to $\sqrt {\frac {3} {\tau (\tau +1) (2 \tau +2)}} \cdot \sigma $.

In comparison, if we aim to detect the presence of spikes in the recorded neural activity data a simple way is to compare the value of the recording to the local average value of the recordings, and conclude the presence of the spike if the difference between the compared values is sufficiently large. In this case the comparison is based on the local average activity value

15 $$ \bar{x_{t}} =\frac{1}{2 \tau +1} \cdot \sum\limits_{u =t -\tau}^{t +\tau} x_{u} $$

for which the contained additive noise has zero mean and a standard deviation equal to $\sqrt {\frac {1}{(2 \tau +1)}} \cdot \sigma $. Consequently, the likely errors affecting this approach will be larger than the estimation errors affecting our proposed methodology since $\sqrt {\frac {3}{\tau (\tau +1) (2 \tau +1)}} \cdot \sigma <\sqrt {\frac {1}{(2 \tau +1)}} \cdot $ for *τ*>1.

## Application

**Fig. 5:**
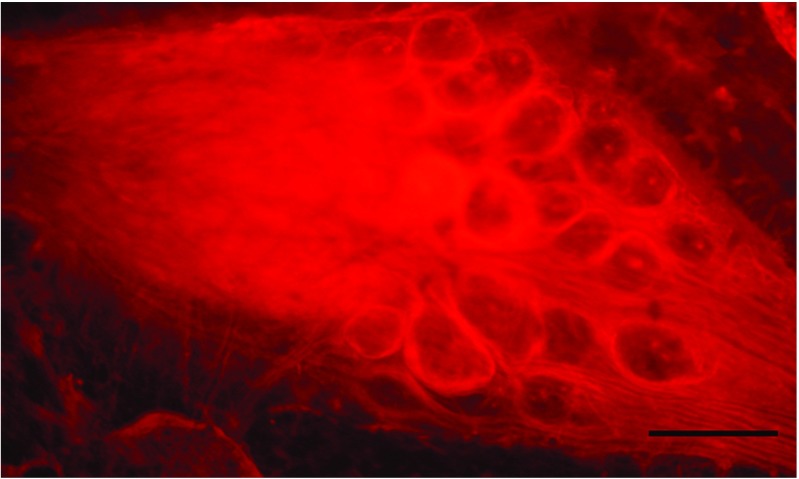
Typical arrangement of neurons in the crab STG. The neuropil is on the *left* side of the image, the neurons (circular shaped surrounds) are arranged in a semi-circle on the *right*. The scale bar is 100 microns

The crustacean stomatogastric ganglion (STG) is one of the most researched neural systems, which is relatively isolated and is responsible for the relatively autonomous delivery of a set of motor functionalities (Harris-Warrick et al. 1992). In the case of brown crabs (*Cancer pagurus*) the STG has 26 neurons organised mainly into two central pattern generator circuits that generate the motor control of the gastric mill and of the pylorus within the foregut of the crab gastric system (Harris-Warrick et al. 1992). The neurons of the crab STG have been studied in detail, their anatomical connectivity, neurotransmitters, response to neuromodulators and other anatomical and functional features are known (Harris-Warrick et al. 1992). A particular feature of the crab STG is that the neuron cell bodies are arranged in a crescent around the neuropil which contains the dendrites and axons of STG neurons and of other neurons as well from higher ganglia that control the STG directly and through neuromodulation. Figure 5 shows a typical arrangement of a crab STG.

**Fig. 6:**
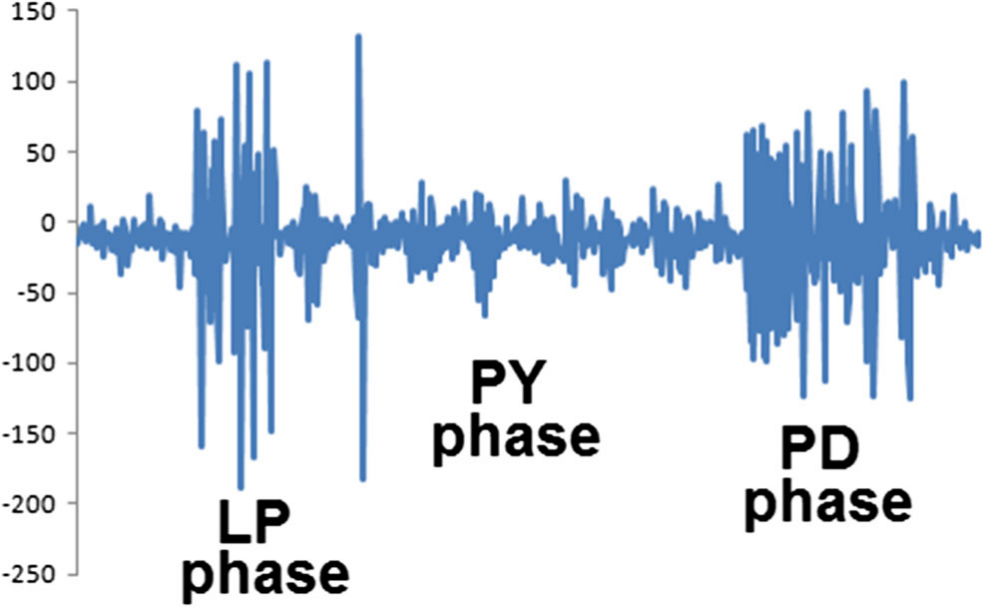
An example of the pyloric rhythm recorded on the lvn. Horizontal axis is time, vertical axis in voltage in arbitrary units

Here we worked on the pyloric circuit within the crab STG. This includes the AB (anterior burster) and two PD (pyloric dilator) neurons, which form the autonomous core oscillator of the network; the LP (lateral pyloric), VD (ventricular dilator), and IC (inferior cardiac) neurons and four or five PY (pyloric constrictor) neurons, which are inhibited by the PD neurons and the AB neuron; the LP neuron inhibits the VD, PD and PY neurons; the PY neurons inhibit the LP and IC neurons; the VD neuron inhibits the LP, IC and PY neurons; and the IC neuron inhibits the VD neuron. There are also electric couplings between the two PD neurons, the PY neurons, the LP and PY, the PD and VD, and the AB and VD neurons - with the exception of PD - PD coupling the others are rectifying electric couplings (Harris-Warrick et al. 1992). The PY neurons fire during the pyloric rhythm normally after the LP neuron and before the PD neurons constituting the PY phase of the rhythm - see Fig. 6. There may be some overlap of PY firing activity with the firing of LP and also PD neurons. The spiking activity of PY neurons is usually tightly synchronised such that the activities of distinct PY neurons may not be easily identifiable in the extracellular recordings from nerves (e.g. lateral ventricular nerve (lvn)). There is some variability in the activity pattern of PY neurons, some having a steep rise while others have a less steep rise of the membrane potential to the level of the activity plateau where the spiking of these neurons happens. Some classify the PY neurons on this basis early and late PYs (EPY and LPY) (Harris-Warrick et al. 1995), while others suggest that this behaviour of PY neurons is gradual and there is no particular distinction between quickly and slowly rising PYs.

Our hypothesis is that under the impact of dopamine PY neurons are de-synchronised (Johnson *et al*. 1993, 1994; Ayali *et al*. 1998). This is expected due to the changes of the strengths of the synapses through which they receive inputs from other neurons (LP and PD) and the reduction of the conductance of the gap junction connections between the PY neurons, which are assumed to contribute to their synchronised activity.

The planar arrangement of the cell bodies of STG neurons makes this neural system particularly well suited for the recording of multiple neurons using voltage-sensitive dye imaging. Following the usual preparation of the crab STG (Gutierrez and Grashow 2009) we either filled with dye identified PY neurons (Stein et al. 2011) or applied the voltage-sensitive dye as a bath solution to the whole de-sheathed ganglion (Städele et al. 2012). PY neurons were identified in both cases on the basis of the analysis of their activity pattern relative to the pyloric rhythm to which these neurons contribute. In the case of dye-filled neurons we used the intracellular electrode recordings of neurons and the recordings from the lvn to identify PY cells, which were filled consequently with dye. In the case of the bath application of the dye we used event-triggered averaging of the recordings to identify the PY neurons. To calculate the event-triggered averaged data we determined manually the beginning of the LP phase of the pyloric rhythm cycles (these are the trigger events) and averaged the imaging data corresponding to identified neurons over all considered consecutive triplets of pyloric rhythm cycles. We applied the same averaging to the data recorded from the lvn as well. The event-triggered averaged imaging data allows robust identification of PY neurons in the case of bath application of the dye. In all experiments we identified three PY neurons in the STGs. Following the dye loading the STG was imaged using a SciMedia MiCAM 02 imaging system (SciMedia, Tokyo, Japan). The imaging data was collected with 1.5ms temporal resolution (i.e. 666 images per second) and each neuron was covered by at least 10 pixels in the imaging data. The raw imaging data was smoothed using a 10 time step sliding window by averaging the raw recordings within the time window. First we imaged the STG in normal saline. For the purpose of dopamine exposure the STG was perfused with saline containing dopamine. We used saline containing 10^−4^ molar concentration dopamine and exposed the STG to this for 20 minutes. The imaging of the dopamine induce state was done after this exposure while still maintaining the perfusion with dopamine containing saline. The raw imaging data was smoothed using a 10 time step sliding window by averaging the raw recordings within the time window.

**Fig. 7:**
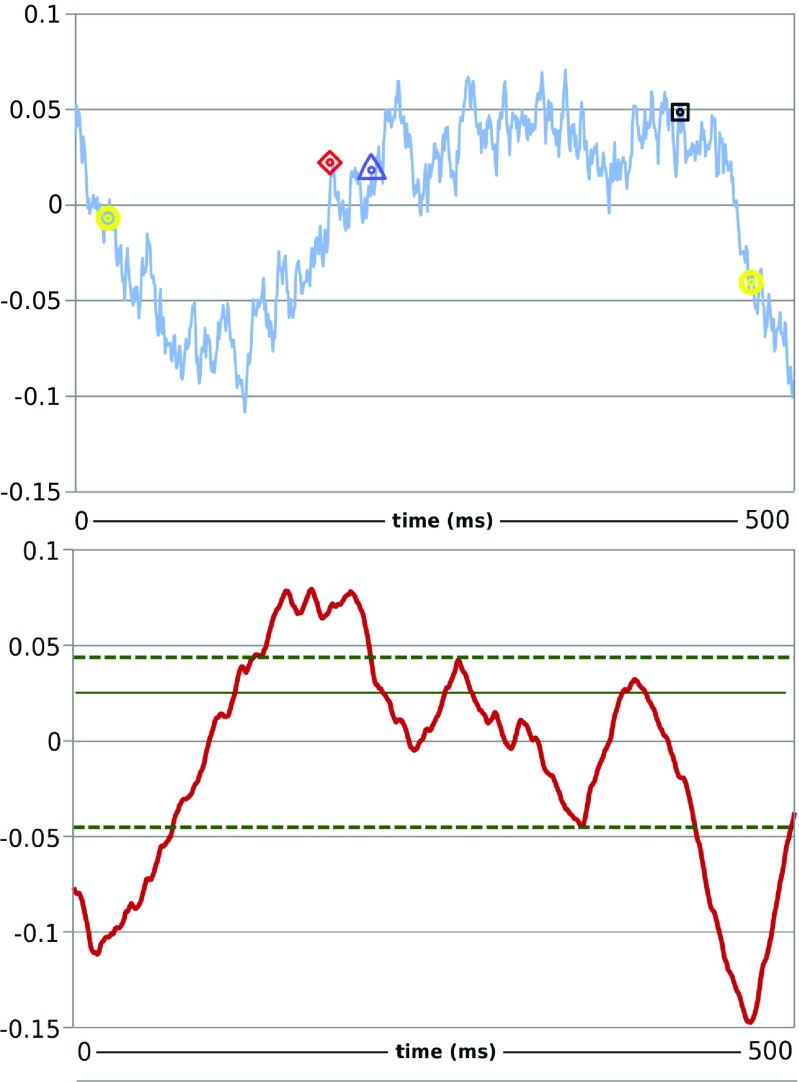
**a** VSD recording of a PY neuron together with the minimum (*yellow circle*) and maximum slope (*red diamond*) points and beginning (*blue triangle*) and end (*black square*) points of the activity plateau determined from the data; **b** The calculated local slope values, the *green horizontal lines* indicate the band of values considered to correspond to the activity plateau following the maximum local slope point. The horizontal axis is time in both cases and the vertical axis is voltage in arbitrary units in **a)** and the local slope value in **b)**

**Fig. 8:**
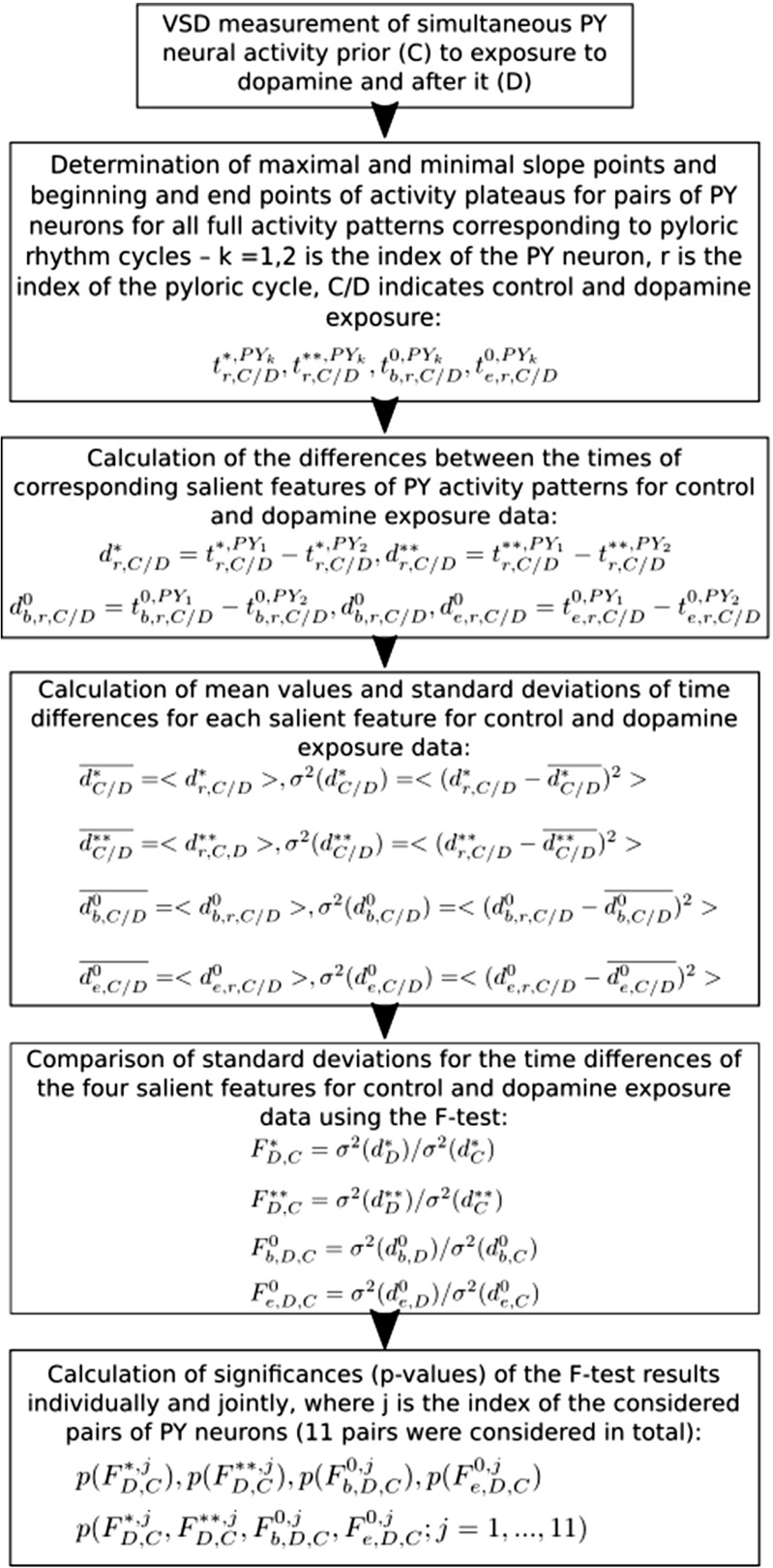
The complete workflow of the data analysis presented in Section 4

**Fig. 9:**
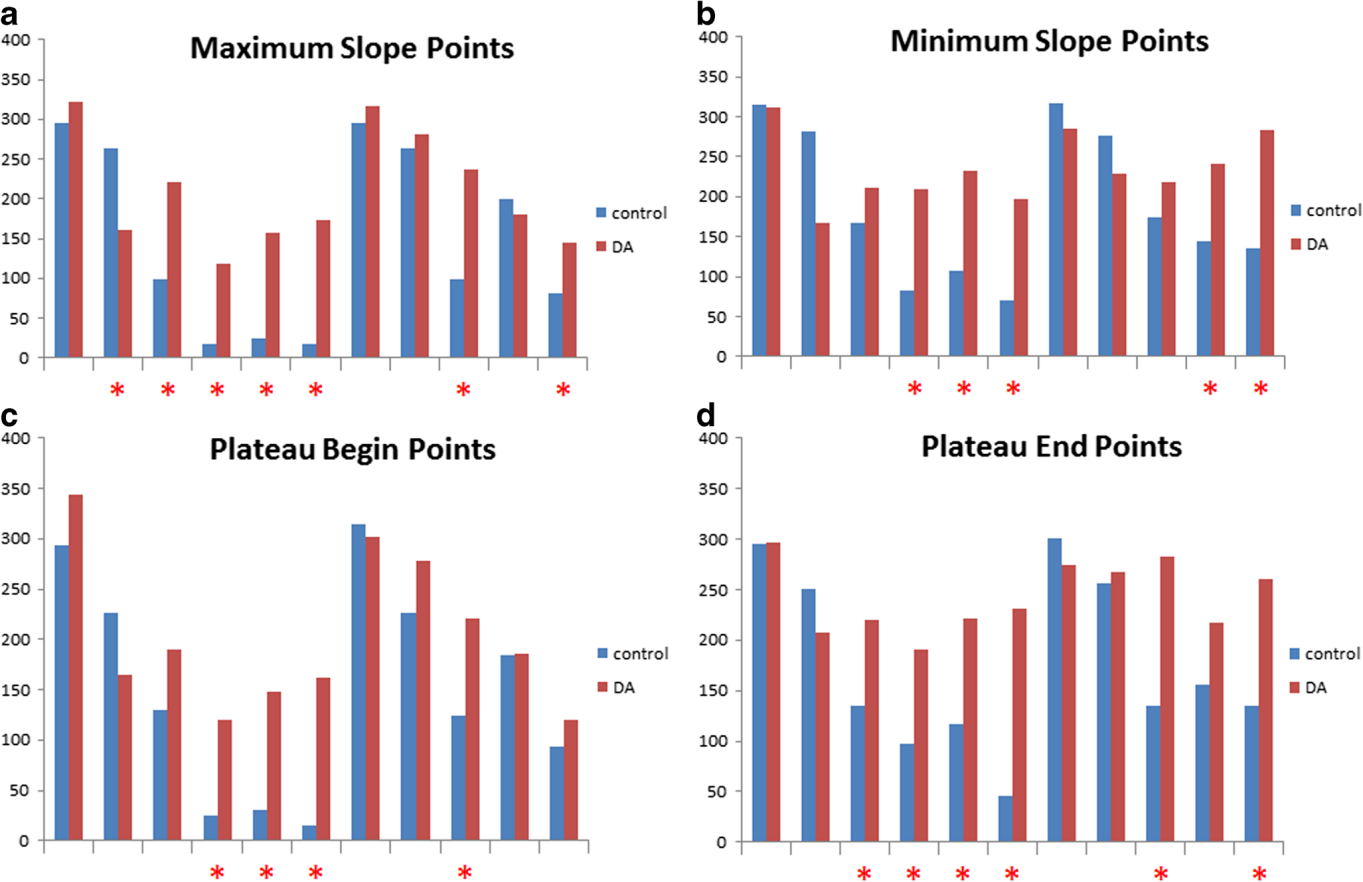
The results of the dopamine experiments. The calculated standard deviation values are shown on the vertical axes. Each pair of bars represents a comparison of a pair of PY neurons. Control indicates standard deviation values calculated before the exposure to dopamine, DA indicates standard deviation values calculated following the exposure to dopamine. The difference is considered statistically significant if the p-value is less than 0.05. Asterisks indicate pairs where the difference is statistically significant

Here we demonstrate the proposed methodology by analysing data recorded from dyed PY neurons in a crab STG. For each PY cell the recordings contained between 50 to 80 full activity patterns, each corresponding to a pyloric rhythm cycle. Figure 7 shows a sample of the recordings including the identified maximum and minimum slope points and beginning and end points of activity plateaus for the recorded PY neurons. To quantify the effect of dopamine on the synchronisation of the PY neurons we measured the temporal delays between corresponding maximum and minimum slope points and beginning and end points of activity plateaus of pairs of PY neurons. In total we considered 11 pairs of PY neurons from four STG preps (two using dye filling and two using bath application of the dye, see Table 1). We calculated the mean values and standard deviations of the temporal delays. We found that the standard deviation of the temporal delays did change significantly in half of the cases (according to the F-test with significance level *p* = 0.05) following the application of the dopamine containing saline. Figure 8 shows the complete workflow of the analysis. Results are shown in Table 2 and Fig. 9. In 22 cases out of 44 comparisons of standard deviations we found that the standard deviations are significantly larger following the effect of the dopamine on the neurons. In one case we found that the calculated standard deviation was significantly lower following the dopamine exposure, and in the remaining 21 cases the difference between the standard deviations was not statistically significant. This means that the exposure to dopamine increased the standard deviation of temporal differences between the corresponding maximum and minimum slope points and beginning and end points of activity plateaus of pairs of PY neurons. The increase of the standard deviation of the temporal differences implies reduction of the temporal locking of the PY neurons, or in other words the de-synchronisation of PY neurons.

**Table 1:** Neuron pair comparisons. In total 11 pairs of PY neurons from four STG preparations were considered

Experiment	Comparison 1	Comparison 2	Comparison 3
1 (dye filling)	PY1 with PY2	-	PY2 with PY3
2 (dye filling)	PY1 with PY2	PY1 with PY3	PY2 with PY3
3 (bath application)	PY1 with PY2	PY1 with PY3	PY2 with PY3
4 (bath application)	PY1 with PY2	PY1 with PY3	PY2 with PY3

Two experiments used dye filling and two were bath applications. For each experiment the neurons were compared under control and DA conditions. Using the F-test a p-value was obtained for each of the four features, thus giving the 44 values as in Table 2

**Table 2:** Results of the DA experiments

Maximum	Minimum	Plateau	Plateau
Slope points	Slope points	Begin points	End points
0.657721015	0.936337338	0.417005168	0.986805014
**0.010577057**	0.064029034	0.097578139	0.317799703
**0.000196868**	0.303675632	0.066103149	**0.019539292**
**2** **.** **6** **6** **5** **5** **0**x**1**0^−19^	**4** **.** **1** **0** **2** **9** **9**x**1**0^−06^	**2** **.** **0** **1** **9** **5** **7**x**1**0^−15^	**0.000686408**
**7** **.** **8** **6** **8** **9** **6**x**1**0^−19^	**9** **.** **6** **9** **1** **4** **8**x**1**0^−05^	**2** **.** **9** **3** **3** **8** **2**x**1**0^−15^	**0.001116038**
**2** **.** **9** **2** **3** **3** **9**x**1**0^−26^	**8** **.** **0** **4** **2** **7** **1**x**1**0^−09^	**8** **.** **0** **7** **8** **0** **4**x**1**0^−27^	**3** **.** **3** **0** **6** **6** **5**x**1**0^−15^
0.729215365	0.637028887	0.841677533	0.687908256
0.734146578	0.411370955	0.302637642	0.819808126
**6** **.** **6** **7** **4** **3** **6**x**1**0^−05^	0.263113108	**0.006803160**	**0.000531943**
0.665538015	**0.024729645**	0.976871651	0.131110278
**0.018054682**	**0.002703960**	0.285120361	**0.004076710**

p-values of the F-test comparisons of the temporal delay standard deviations. Significant values are shown in bold

Considering a *p* = 0.05 significance level, we would expect 95 % of the comparison cases to lead to a non-significant result, and only 5 % of the cases to show a significant change in the variance values (i.e. 2.5 % would have significantly lower and 2.5 % would have significantly higher variances). For our comparisons that would mean that 42 of the 44 comparisons would show no significant difference, one case would show the the post-DA variance is significantly larger and one case would show that the post-DA variance is significantly lower than the pre-DA variance. In contrast, our results showed 22 cases presenting significantly larger post-DA variances, 21 cases showing no significant change and one case showing a significantly lower post-DA variance. To put this more formally, assuming independence of the comparisons, we can calculate the overall p-value of the combined results of the experiments, which is 4.85 × 10^−5^, indica ting that the our result about the increase in the variances is indeed statistically significant. The results thus confirm our hypothesis about the de-synchronisation effect of dopamine exposure on PY neurons.

Our approach offers a way to quantify the extent of de-synchronisation of PY neurons in response to exposure to dopamine. The presented analysis of PY neurons demonstrates that the methodology that we proposed can be applied successfully to analyse the dynamics of temporal relationships of neural activities using optical imaging data.

## Discussion and conclusions

It is of vital importance that neural circuits are adaptive and flexible in the delivery of their functionality. Such flexibility relies on the dynamics of the temporal relationship between the neurons forming those neural circuits. The recording of many synaptically connected neurons, at individual neuron resolution, has not been possible under physiologically realistic conditions until relatively recently. However, current optical recording techniques using voltage sensitive dyes and calcium dyes allow high spatio-temporal resolution recordings to be made of many neurons. Such techniques enable us to study the dynamics of temporal relationships of neural activities in biological neural circuits.

We propose here a method for the analysis of such optical data for understanding the dynamics of the temporal relationship of the activities of individual neurons. The proposed method relies on the robust identification of salient points of the activity patterns of individual neurons, such as the minimum and maximum slope points and the beginning and end points of depolarisation plateaus (the latter two only in appropriate cases). The method is very important because it allows robust analysis of optical neuro-imaging data to determine activity phases of neurons and on the basis of this allows the quantification and analysis of the dynamics of activity patterns of multiple neurons. As we have shown in Section 3, other methods based on the calculation of average measurements are less robust than the method proposed by us. This kind of analysis is key for the understanding of the emergent functionality of neural systems. Consequently the method that we propose here improves the reliability of the use of optical imaging data for this kind of analysis.

We applied the proposed method of analysis to neurons recorded in the crab STG and it was shown that, as expected, there is a statistically significant, measurable desynchronisation effect of DA on the considered PY neurons. This is the first time to show this effect in a physiologically realistic setting of the STG, i.e. previous measurements implying this result were made in the presence of neurotoxic substances to achieve pharmacological isolation of neurons.

As we noted above the method that we described is expected to work at best in the case of neurons for which the depolarisation plateau means a larger change in the recorded membrane potential than the spikes themselves. However, we also expect that the method should work well even for neurons where this is not the case (e.g. neurons of the mammalian cortex). In the case of these neurons the determination of minimum and maximum slope points is feasible and these allow the robust measurement of the dynamics of the temporal relationships of the activity patterns of these neurons using optical imaging data. Sufficiently fast temporal resolution and sufficiently fine spatial resolution should allow the proposed method to be applied to VSD imaging data from cortical slices or from in-vivo neural resolution VSD imaging of neural activity inside of the brain. The proposed approach is likely to provide a better understanding of the fine temporal dynamics of the activity of multiple neurons than for example Calcium imaging data, which is less noisy but operates on a longer time scale.

The method that we propose works off-line as we indicated above. However, it is possible at least in principle to extend it to on-line application, if the proposed analysis method is integrated with the recording of the data. Having sufficiently fast processors this should not represent a major technical challenge. If the method is applied on-line its application is only limited by the time window required for the calculations (consider in particular the case of forward slope calculation), however even this constraint can be mitigated by considering a predictive application of the methodology (e.g. predicting the timing of salient points on the basis of previously determined salient points and correcting the predictions when the required data becomes available). This kind of on-line application of the methodology would allow setting of additional stimulation of selected neurons depending on the activity pattern phase of measured neurons. This would make the design of more elaborated experiments, involving measurement and experimental modulation of the activity of multiple neurons, possible.

The method that we described in this paper is also applicable to other kinds of noisy biological recordings where robust quantification of key transitions and the measurement of relative transition dynamics across multiple processes is required. As we have shown, the calculation of local slope values is more robust than the calculation of usual averages and this difference in robustness of calculations may be critically important in the context of estimation of activity pattern features from noisy recordings. For example, such cases may include other neural systems (e.g. phase determination of swim pattern generators in leeches or snails) or recordings from muscles (e.g. heart muscles or muscles involved in rhythmic movement or swimming).

The Delphi code used for the analysis in this paper has been made available for download in BitBucket at https://bitbucket.org/jannetta/temporal-dynamics.
